# Albumin-EDTA-Vanadium Is a Powerful Anti-Proliferative Agent, Following Entrance into Glioma Cells via Caveolae-Mediated Endocytosis

**DOI:** 10.3390/pharmaceutics13101557

**Published:** 2021-09-25

**Authors:** Itzik Cooper, Orly Ravid, Daniel Rand, Dana Atrakchi, Chen Shemesh, Yael Bresler, Gili Ben-Nissan, Michal Sharon, Mati Fridkin, Yoram Shechter

**Affiliations:** 1 The Joseph Sagol Neuroscience Center, Sheba Medical Center, Ramat-Gan 52620, Israel; Orly.ravid@sheba.health.gov.il (O.R.); Daniel.Rand@sheba.health.gov.il (D.R.); Dana.Atrakchi@sheba.health.gov.il (D.A.); Chen.Shemesh@sheba.health.gov.il (C.S.); Yael.Bresler@sheba.health.gov.il (Y.B.); 2 School of Psychology, The Reichman University, Herzliya 4610101, Israel; 3 The Nehemia Rubin Excellence in Biomedical Research, The TELEM Program, Sheba Medical Center, Tel-Hashomer, Ramat Gan 52620, Israel; 4 Sackler Faculty of Medicine, Tel-Aviv University, Tel-Aviv 69978, Israel; 5 Department of Biomolecular Sciences, The Weizmann Institute of Science, Rehovot 76100, Israel; Gili.Ben-nissan@weizmann.ac.il (G.B.-N.); michal.sharon@weizmann.ac.il (M.S.); yoram.shechter@weizmann.ac.il (Y.S.); 6 Department of Organic Chemistry, The Weizmann Institute of Science, Rehovot 76100, Israel; mati.fridkin@weizmann.ac.il

**Keywords:** albumin, conjugates, vanadium, cancer, prodrug

## Abstract

Human serum albumin (HSA) is efficiently taken up by cancer cells as a source of carbon and energy. In this study, we prepared a monomodified derivative of HSA covalently linked to an EDTA derivative and investigated its efficacy to shuttle weakly anti-proliferative EDTA associating ligands such as vanadium, into a cancer cell line. HSA-S-MAL-(CH_2_)_2_-NH-CO-EDTA was found to associate both with the vanadium anion (+5) and the vanadium cation (+4) with more than thrice the associating affinity of those ligands toward EDTA. Both conjugates internalized into glioma tumor cell line via caveolae-mediated endocytosis pathway and showed potent anti-proliferative capacities. IC_50_ values were in the range of 0.2 to 0.3 µM, potentiating the anti-proliferative efficacies of vanadium (+4) and vanadium (+5) twenty to thirty fold, respectively. HSA-EDTA-VO^++^ in particular is a cancer permeable prodrug conjugate. The associated vanadium (+4) is not released, nor is it active anti-proliferatively prior to its engagement with the cancerous cells. The bound vanadium (+4) dissociates from the conjugate under acidic conditions with half maximal value at pH 5.8. In conclusion, the anti-proliferative activity feature of vanadium can be amplified and directed toward a cancer cell line. This is accomplished using a specially designed HSA-EDTA-shuttling vehicle, enabling vanadium to be anti-proliferatively active at the low micromolar range of concentration.

## 1. Introduction

Intensive studies have been carried out on the insulin-like effects of vanadium salts. Vanadium mimics the action of insulin in insulin responsive tissues and in diabetic rodents via insulin-independent pathways [1,2,3], which is reviewed in [4,5]. Vanadium belongs to a family of metals, which interferes with cellular redox homeostasis [6], and as such was investigated also for its anti-cancer efficacy. Vanadium is an element with a wide range of effects on the mammalian organism. In recent years, many studies were published regarding its various organic complexes in view of their application in medicine and the fact that the bioactive complexes/compounds of this metal can be therapeutically active at low concentrations [7]. With its physiological duality, vanadium is essential in trace amounts and toxic at concentrations above 10 µM. Its biological activities include anti-viral, anti-bacterial, anti-parasitic, anti-fungal, anti-cancer, anti-diabetic, anti-hypercholesterolemic, cardio-protective, and neuroprotective activity [8]. Moreover, in vivo studies reported chemo-preventive effects of vanadium complexes, whereas observations regarding therapeutic activities were limited [9,10,11,12,13,14]. Indeed vanadium can act in two opposing directions: as a metabolic factor, it might promote proliferation, and on the other hand due to its ability to generate ROS and/or to inhibit a large variety of phosphatases and hydrolases, it can act as an anticancer agent [6]. Consequently, the question arose whether these two opposing effects can be dissociated to permit conversion of vanadium exclusively into an anticancer agent.

In this study, we initially turned vanadium into a prodrug, capable of uptake preferentially by cancerous cells. A derivative of EDTA was covalently linked to HSA in a monomodified fashion. Albumin is largely taken by malignant tissues as a source of carbon and energy [15]. Albumin is also a natural transport protein with long circulatory half-life, which promotes it as an attractive candidate for half-life extension and targeted intracellular delivery of drugs attached by covalent conjugation or association [16].

Both vanadium (+4) and vanadium (+5) associate with EDTA at physiological pH [17]. We assumed that the resultant HSA- EDTA-vanadium conjugates will be inactive extracellularly, but will release bound vanadium both in the cytosol and even more efficiently at the acidic pH of the lysosome, following internalization.

Here, we wished to determine whether vanadium (+4) or vanadium (+5) generate intracellular cytotoxicity, if shuttled into a cancerous cell line with this HSA-EDTA carrier. Likewise, we attempted to identify conditions that eliminate the proliferative effects of vanadium and preserve solely its anti-proliferative efficacy. Our efforts in those directions are presented here in detail.

## 2. Materials and Methods

### 2.1. Materials

Dulbecco’s Modified Earl’s medium (DMEM) was purchased from Gibco (Life Technologies, Carlsbad, CA, USA). Gentamicin, glutamine, fetal calf serum (FCS), and penicillin/streptomycin were obtained from Biological Industries (Kibbutz Beit Haemek, Galilee, Israel). Human serum albumin (HSA), diethylenetriamine pentaacetic dianhydride (EDTA-dianhyride), *N*(2-aminethyl) malemide, 4.4′ dithiodipyridine (4.4DTDP), dithiothreitol (DTT), phosphatase acid from potato (#P-3762), pNitrophenyl phosphate (pNPP), phenylarsine oxide (PAO), indomethacin (IND), nystatin, methyl β cyclodextrin (MCD), and bafilomycin A1 (BAF) were purchased from Sigma Aldrich (Jerusalem, Israel). Sodium metavanadate (NaVO_3_) and Vanadyl chloride (VOCl_2_) were from BDH Chemicals Ltd. Poole England. Ethylenediaminetetraacetic acid (EDTA) from Baker Analyzed A.C.S Reagents, and PEG_30_-SH (M-SH-30K) were purchased from Jenkem Technology (Plano, TX, USA). All other materials used in this study were of analytical grade.

### 2.2. Preparation of Mercaptoalbumin

About one third of cysteine-34 of HSA is disulfide-bonded to glutathione or cysteine and this can be reversed by mild reduction with dithioerithritol [18]. This allows us to obtain conjugates containing 0.56 mole EDTA per mole of human serum albumin (Supplementary Table S1). HSA (1.4 g, 20 µmol) dissolved in 0.1 M Hepes buffer (pH 7.3) followed by the addition of one equivalent of dithiothreitol (20 µmol). The reaction was carried out for 1 h at 0 °C and dialyzed over a period of two days with several changes of H_2_O and lyophilized. This procedure removes mixed disulfide bonded glutathione or cystine from the cysteinyl moiety of HSA [19]. Mercapto-HSA prepared by this procedure contains 0.7 + 0.05 mole-SH per mole human serum albumin, as determined with 4.4 dithiodipyridine (4.4′ DTDP) using ε_324_ = 19,800 [20].

### 2.3. Preparation of EDTA-NH-(CH_2_)_2_-Maleimide (EDTA-Maleimide)

EDTA-Dianhydride (38 mg, 100 µmol) suspended in 1.0 mL DMSO and transferred to a tube containing 110 µmol (27 mg) MAL-(CH_2_)_2_-NH_2_. DIPEA (*N*,*N*-Disopropylethylamine) was then added in aliquots to achieve neutral pH value, upon 100 times dilution of aliquots in H_2_O. Following one hour, the product EDTA-maleimide was obtained by centrifugation, washed twice with DMSO and stored at −70 °C until used.

### 2.4. Preparation of HSA-S-MAL-EDTA

*N*-(2 aminoethyl) maleimide (2.5 mg, 10 µmol) was dissolved in 1.0 mL of 1 M Hepes buffer (pH 7.3) and transferred immediately to a glass tube containing 38 mg (100 µmol) of EDTA-dianhydride. The reaction mixture was stirred for 30 min and combined with a solution of Mercapto-HSA (210 mg/3.0 mL H_2_O, 3 µmol). Following 1 h, the product (HSA-S-MAL-(CH_2_)_2_-NH-CO-EDTA) was dialyzed against H_2_O over a period of three days with several changes of H_2_O and lyophilized.

### 2.5. Characterization of HSA-S-MAL-(CH_2_)_2_-NHCO-EDTA by Reversed Phase Liquid Chromatography, Coupled to Mass Spectrometry (LC-MS) Analysis

The different protein samples were diluted to 0.17 µM in 100 mM ammonium acetate, pH 6.8. A total of 5 µL from each sample were loaded onto a monolithic reversed phase column [21] and eluted over a gradient of 10–60% acetonitrile, during 15 min, at column temperature of 60 °C. The HSA proteins eluted at 43% acetonitrile and were directly sprayed into a modified Q Exactive Plus EMR Orbitrap mass spectrometer [21], for intact mass measurements. The instrument was operated using the HESI source, at a flow rate of 15 µL/min, using sheath gas 10 and auxiliary gas 3. The inlet capillary was set to 320 °C, capillary voltage 4.3 kV, fore vacuum pressure 1.54 mbar, and trapping gas pressure 0.8, corresponding to HV pressure of 3.0 × 10^−5^ mbar and UHV pressure of 2.2 × 10^−10^ mbar. The source was operated at a constant energy of 2 V in the flatapole bias and interflatapole lens. Bent flatapole DC bias and gradient were set to 1.7 and 10 V, respectively, and the HCD cell was operated at 15 V. Measurements were performed at inject time of 250 and resolution of 10,000. Masses were calculated by the computational suite UniDec v. 4.1.1 [22] (2019, University of Arizona, Tucson, AZ, USA).

### 2.6. Evaluating the Affinities of HSA-EDTA to Vanadium: Reversal of Vanadium-Evoked Inhibition of Acid Phosphatase

This assay was carried out essentially according to reference [4] with slight modifications. It evaluates the efficacy of vanadium chelators toward vanadium, by determining their potency to reverse vanadium-evoked inhibition of acid phosphatase at pH 7.3. Each tube contains 0.5 mL of 0.05 M Hepes buffer pH 7.3, 1.0 M KCl, p-nitophenylphosphate, (0.2 mM) either NaVO_3_ or VOCl_2_ (5 µM), increasing concentration of the studied chelator and acid phosphatase (50 µg/tube). Following 40 min at 25 °C, NaOH (20 µL from 4 M NaOH) was added and the absorbance corresponding to the formed p-nitrophenolate was determined at 410 nm. IC_50_ is defined here as the concentration of the vanadium chelator that reversed half maximally vanadium (+4) or vanadium (+5) evoked inhibition of acid phosphatase. It should be noticed that since this is an in-direct method, direct measurements of released vanadium using procedures such as ICP-MS, should be conducted in future studies.

### 2.7. Preparation of HSA-EDTA-Vanadium

HSA-EDTA (25 mg, 0.37 µmol) dissolved in 0.2 mL H_2_O and VOCl_2_, or NaVO_3_, 4 molar excess was then added. The reaction mixture was loaded on a Sephadex G-50 column (12 × 1.7 cm) pre-equilibrated and run with 0.01 M NaHCO_3_ (pH 8.22). The peak corresponding to the protein fraction was pooled and lyophilized.

### 2.8. Preparation of Rhodamine-Labeled HSA-EDTA

Rhodamine-labeled HSA and HSA-EDTA were prepared by dissolving 17 mg of each (~0.25 µmol) in 0.2 mL of 0.1 M Na_2_CO_3_ (pH 10.3). Rhodamine B isothiocyanate 0.9 mg (2.5 molar excess over HSA) was then added and the reaction was carried out for 1 h at 25 °C. The reaction mixture was loaded on a Sephadex G-50 column (1.7 × 14 cm) equilibrated and run in the same buffer. The tubes containing rhodamine-labeled HSA were pooled, dialyzed against water, and lyophilized. Rhodamine-HSA and rhodamine-HSA-EDTA prepared by this procedure contain 0.95 ± 0.1 mole of rhodamine/mole of HSA as determined by its absorbance at 550 nm using ε_550_ = 11,400.

### 2.9. Growth Inhibitory Effects of EDTA and Vanadium Containing Conjugates

The glioma cell line CNS-1 (obtained from Mariano S. Viapiano [23]) was grown in 96 well plates in DMEM containing 10% fetal calf serum; 2 mM L-glutamine, penicillin (100 units/mL), and streptomycin (0.1 mg/mL) under humidified atmosphere containing 5% CO_2_. Cells were seeded at 1000 cells/well. Twenty-four hours later, the EDTA and vanadium containing conjugates were added to each plate to give concentrations as indicated in the text. Control experiments using non-cancer cells were conducted with primary bovine brain pericytes and CD34+ human endothelial cells (both obtained and characterized at the Artois University, France [24,25,26]) treated with the HSA-EDTA-VO^++^ conjugate. These cells were seeded at 15,000 cells/well in ECM medium (Sciencell, Carlsbad, CA, USA), which was composed as follows: 5% fetal calf serum (Gibco, Gaithersburg, MD, USA), ECGS supplements, and 50 mg/mL gentamicin (Biological industries, Beit-Haemek, Israel). Cells were treated the day after. Cell viability was measured after 72 h using a standard MTT (3-(4,5-dimethylthiazol-2-yl)-2,5-diphenyltetrazolium bromide) assay as described before [27]. Experiments were repeated at least 3 times in quadruplicate. IC_50_ values were calculated from the dose response curves using a median-effect plot.

### 2.10. Immunocytochemistry

CNS-1 cells (60,000/well) were seeded on cover slips in 24 well-plates. After 24 h, growth medium (10% FCS, penicillin (100 U/mL) streptomycin (0.1 mg/mL) and L-glutamine (2 mmol/L) dissolved in DMEM was replaced with fresh medium containing 5 µM rhodamine-labeled HSA or HSA-EDTA-VO^++^. After 5 min, 1 h, or 24 h, the cells were washed with cold PBS and fixed with 4% paraformaldehyde for 15 min. The cells were then stained with Alexa fluor 488-phalloidin (Thermo Fisher Scientific, Waltham, MA, USA) for 20 min and 2 min with Hoechst (Sigma, Burlington, MA, USA). Cells were rinsed with PBS and coverslips were mounted and observed with Olympus IX43 fluorescence microscope.

### 2.11. Uptake of Rhodamine-HSA and Rhodamine-HSA-EDTA-VO^++^

CNS-1 cells (50,000/well) were seeded in 24 well plates. After 48 h, cells were washed with 37 °C phosphate buffer saline (PBS) and pre-incubated in the absence or presence of different blockers in serum-free medium. Pre-incubation conditions of the different blockers were as follows: PAO (clathrin-mediated endocytosis inhibitor, 3 µM) and BAF (metabolic inhibitor, 100 nM) were added only during the pre-incubation period for 30 min. The caveolae-mediated endocytosis inhibitors MCD (5 mM), nystatin (54 µM), and IND (100 µM) were added for 10 min at the pre-incubation period and also during the uptake. After pre-incubation, the cells were incubated with rhodamine-labeled HSA or HSA-EDTA-VO^++^ (0.25 µM) with or without the blockers for 1 h at 37 °C. Cells were then rinsed twice with ice-cold PBS and solubilized with 0.5 M NaOH/0.05% SDS (500 µL/well). A total of 200 µL from each well were transferred to black 96 well plate and the fluorescence was measured using TECAN infinite 200 Pro plate reader at excitation/emission wavelengths of 544/576 nm. A total of 50 µL from each well were evaluated for protein content using a standard BCA assay (Thermo Scientific, Waltham, MA, USA). The effect of the different blockers was calculated after reduction of blanks (the fluorescence of supernatants without rhodamine labeled compounds) and normalization for protein content. Data are presented as the percentage of uptake relative to cells without blockers. 

### 2.12. Statistical Analysis

Statistical analyses were performed using the Prism 6 software. Data are presented as the means ± standard error of the mean (SEM). Differences between two groups were assessed by an unpaired *t*-test and among three or more groups by a one-way analysis of variance followed by Tukey’s Multiple Comparison Test. A *p*-value of less than 0.05 was considered to be statistically significant.

## 3. Results

### 3.1. Preparation of Monomodified HSA-EDTA Derivative

**Figure 1:**
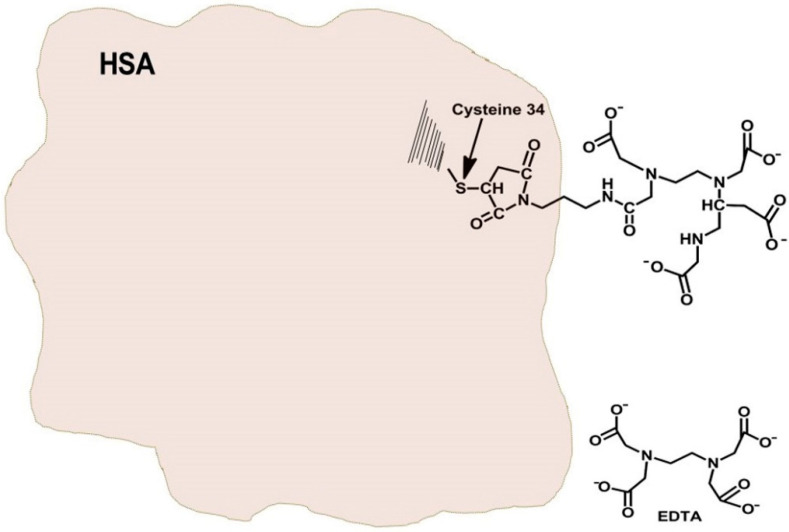
Schematic representation of EDTA and the monomodified HSA-EDTA derivative (HSA-S-MAL-(CH_2_)_2_-NH-CO-EDTA).

HSA contains a single cysteinyl moiety at position 34, and its derivatization has little or no effect on the three-dimensional configuration of this carrier protein [15]. Our initial intention was therefore to obtain a monomodified derivative of HSA, containing a single moiety of EDTA. Since EDTA-dianhydride is insoluble in organic solvents the synthesis was carried out under aqueous conditions in 1.0 M Hepes buffer (pH 7.3) for a period of 30 min. During this period, unreacted EDTA-dianhydride is fully hydrolyzed, avoiding the risk of reacting with the amino side chains of HSA (preliminary observation). MAL-containing compounds lose a significant amount of their alkylating capacity under these conditions [28]; however, a sufficient level of MAL-(CH_2_)_2_-NH-CO-EDTA remained for alkylating the single cysteinyl moiety of HSA. All non-covalently linked low molecular-weight molecules were then removed by extensive dialysis, prior to lyophilization (Experimental part). Figure 1 shows a schematic presentation of EDTA and the monomodified HSA-EDTA derivative (HSA-S-MAL-(CH_2_)_2_-NH-CO-EDTA) prepared.

### 3.2. Characterization of HSA-S-MAL-(CH_2_)_2_-NHCO-EDTA by LC-MS

**Figure 2:**
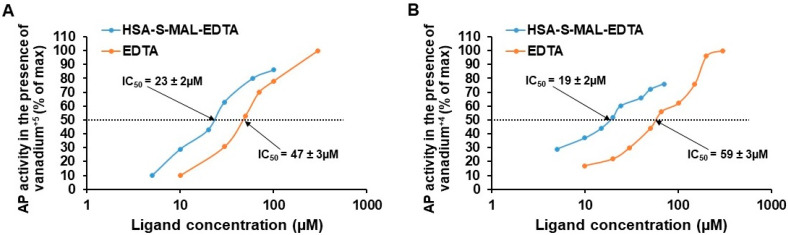
Reversal of inhibition of acid phosphatase (AP) by EDTA and HSA-EDTA at pH 7.3. (**A**) Reversal of NaVO_3_ (+5) evoked inhibition of acid phosphatase and; (**B**) reversal of VOCl_2_ (+4) evoked inhibition of acid phosphatase. Vanadium concentration was 5 µM. AP, acid phosphatase.

This procedure was found particularly suitable for HSA-derivatives, since the first stage (denaturation under acidic conditions at 60 °C) eliminates non-covalent interactions (like binding of long-chain free fatty acids) from the protein. Supplementary Materials Table S1 summarizes the MW of mercapto-HSA and two batches of HSA-S-MAL-(CH_2_)_2_-NHCO-EDTA prepared by us. Interestingly enough, the two batches showed additional masses in the vicinity of 150 Da, rather than 530 Da, which was expected for the covalently linked MAL-(CH_2_)_2_-NHCO-EDTA to HSA. We therefore postulated that the peptide bond connecting HSA to EDTA, namely HSA-MAL-(CH_2_)_2_-NH--CO-EDTA is cleaved during the first stage of the procedure, via a mechanism resembling the hydrolysis of maleyllysine, described by Butler et al. [29]. The MW of the “tail” linked to HSA was calculated to be 156 Da, and additions of 159 and 147 Da were obtained for the two different batches of HSA-S-MAL-(CH_2_)_2_-NHCO-EDTA prepared by us (summarized in Supplementary Table S1). Supplementary Figure S1 shows the deconvoluted mass distribution of mercapto-HSA and of HSA-S-MAL-(CH_2_)_2_-NHCO-EDTA. This analyses suggested that about 56% of the molecules were modified. Cysteine 34 of albumin is known to “resist” derivatization of somewhat larger –SH reagent, due to its orientation in the three dimensional structure of albumin. These analyses also suggested that these conjugates are mono-modified, in spite of the fact that associating affinity towards vanadium was elevated 3–4 times (Figure 2).

### 3.3. Association of Vanadium with HSA-EDTA: Comparison to EDTA and EDTA-Maleimide

Figure 2A shows the reversal of NaVO_3_ (+5) evoked inhibition of acid phosphatase by EDTA, EDTA-maleimide and HSA-EDTA at pH 7.3. Half-maximal values were 47, 56 and 23 µM for EDTA, EDTA-maleimide and HSA-EDTA respectively. Figure 2B demonstrates the reversal of VOCl_2_ (+4) evoked inhibition of acid phosphatase by those ligands. In this case, half maximal values amounted to 59, 71 and 19 µM for EDTA, EDTA-maleimide and HSA-EDTA respectively. Thus the associating affinity toward both forms of this metalooxide increased 3–4 folds (Figure 2) when this chelator is linked to cysteine-34 of this carrier protein. 

### 3.4. Preparation of HSA-EDTA Vanadium Conjugates

**Figure 3:**
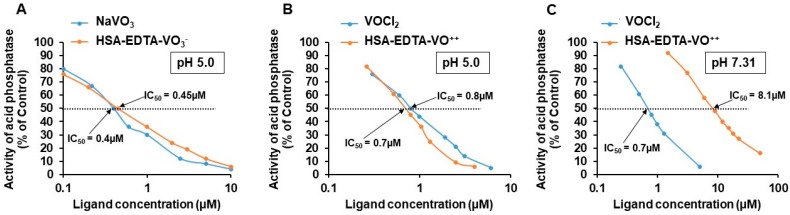
Dose-dependent inhibition of acid phosphatase at pH 5.0 and 7.31 by Sephadex-purified HSA-EDTA-vanadium and the free metalooxides. The incubation assay was run for 40–60 min at 25 °C in tubes (0.6 mL) containing 0.1 M KCl-1 mM HCl (pH 5.0, **A**,**B**) or 0.1 M KCl-100 mM Hepes buffer (pH 7.31, **C**). Each tube contained PNPP (0.2 mM), 5 µg acid phosphatase (**A**,**B**) or 25 µg (**C**) and the indicated concentrations of HSA-EDTA-vanadium or the free metalooxide. The assay was terminated with NaOH, upon reaching OD_410_ = 0.9 ± 0.1 in tubes having no HSA-EDTA-vanadium or the free metalooxide. Results are expressed as the mean ± SEM of three independent experiments.

HSA-S-MAL-EDTA was treated with four-fold molar excess of NaVO_3_ or VOCl_2_ and the resultant conjugates were purified on a Sephadex G-50 column (Experimental procedures). This purification step removed unbound vanadium as well as vanadium molecules adsorbed to HSA in an EDTA-independent fashion (in control experiments we added to native HSA 4-fold molar excess of vanadium, transferred them on the Sephadex G-50 column, pooled and lyophilized the void volume, and examined it for the presence of vanadium by the acid phosphatase assay. No vanadium could be detected). Following gel-filtration, both conjugates contain 0.56 ± 0.005 mole vanadium per mole HSA-EDTA. This was quantitated by determining their dose-dependent inhibitory potencies toward acid-phosphatase at pH 5.0 (Figure 3A,B). At this pH (or lower), vanadium dissociates fully from the conjugates, regaining the efficacy of the free metalooxide to inhibit this enzymatic activity (subsequent paragraph). IC_50_ values were 0.40 ± 0.03 µM for vanadium (+5) and 0.45 ± 0.02 µM for HSA-EDTA-VO_3_^−^ (Figure 3A). Vanadium (+4) and HSA-EDTA-VO^++^ inhibits this enzymatic activity at pH 5.0 with IC_50_ values of 0.8 ± 0.04 and 0.7 ± 0.03 µM respectively (Figure 3B). For comparison, the efficacy of vanadium (+4) and HSA-EDTA-VO^++^ to inhibit acid phosphatase at pH 7.3 is shown in Figure 3C. IC_50_ values amounted to 0.7 ± 0.03 and 8.1 ± 0.3 for vanadium (+4) and HSA-EDTA-VO^++^, respectively.

### 3.5. Stability of HSA-EDTA-VO^++^ as a Function of pH

As shown in Figure 3B, the vanadium (+4) dissociates fully at pH 5.0 from the conjugate, regaining the efficacy of the free metalooxide to inhibit acid-phosphatase. Table 1 summarizes the IC_50_ values for the inhibition of this enzymatic activity at varying pH values. The dissociated fraction of vanadium (+4) from the conjugate as a function of pH was calculated. IC_50_ values varied between 1.0 ± 0.03 µM at pH 5.4 (corresponding to 70% dissociation) to 5 µM at pH 7.15 (corresponding to 14% dissociation). Extrapolation of these values revealed that half maximal dissociation of vanadium (+4) from the conjugate takes place at pH 5.8.

### 3.6. HSA-EDTA-Vanadium Conjugates Are Powerful Anti-Proliferative Agents

**Figure 4:**
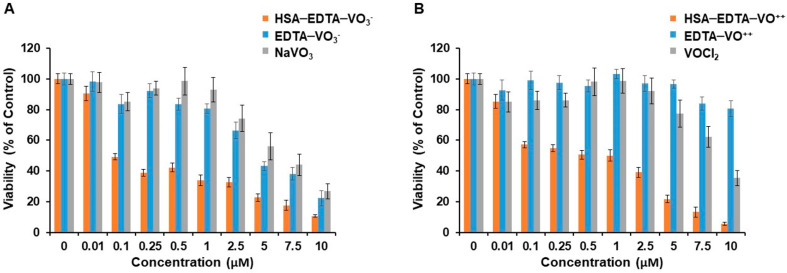
Anti-proliferative efficacies of free metalooxide, 1:1 complexes of EDTA and vanadium, and HSA-EDTA-vanadium conjugates in the CNS-1 glioma cell line. Dose-dependent toxicity experiments were conducted as described in the Section 2. HSA-EDTA-VO_3_^−^, NaVO_3_, and EDTA-VO_3_^−^ (**A**) or HSA-EDTA-VO^++^, VOCl_2_, and EDTA-VO^++^ (**B**) were added to the cell culture for 72 h before MTT toxicity assay was applied to determine their anti-proliferative efficacies. Experiments were repeated at least three times in quadruplicate. Data are presented as the mean percentage ±SEM. *n* = 12 per treatment from at least 3 different experiments.

Figure 4A shows the dose-dependent anti-proliferative efficacy of HSA-EDTA-VO_3_^−^ in the CNS-1 cell line. This was compared to that of free vanadate (+5) and to a 1:1 complex of EDTA with VO_3_^−^. HSA-EDTA-VO_3_^−^ facilitates its anti-proliferative effect with IC_50_ value of 0.27 + 0.03 µM, potentiating the effect of vanadium (+5) about 20 folds (IC_50_ = 5.3 µM, Table 2). The complex of EDTA with vanadium (+5) also facilitates a significant anti-proliferative effect (Table 2), suggesting that this metalooxide can significantly dissociate from EDTA during the three-day period of incubation with the cells. Figure 4B shows the dose-dependent anti-proliferative efficacy of HSA-EDTA-VO^++^ as compared to that of the vanadyl cation and to a 1:1 complex of EDTA with VO^++^. This conjugate was found to be a powerful anti-proliferative agent as well (IC_50_ = 0.34 ± 0.03 µM, Table 2). It potentiated the effect of vanadyl about 26 times. (IC_50_ = 8.9 µM). Unlike EDTA-VO_3_^−^ (Figure 4A), the one to one complex EDTA-VO^++^ had negligible anti-proliferative efficacy at concentrations above 5 µM (Figure 4B, Table 2). Thus, HSA-EDTA-VO^++^ appears to be a ‘silent’ prodrug prior of engagement with the CNS-1 cells, where a powerful anti-proliferative effect is developed. Neither one of the three components comprising HSA-EDTA, showed anti-proliferative efficacy with IC_50_ lower than 10 µM (Table 2). The anti-proliferative effect of the HSA-EDTA-VO^++^ conjugate was examined also in non-cancer cells (primary bovine brain pericytes and CD34+ human endothelial cells) and the potency towards these cells was found to be much lower than towards the CNS-1 glioma cells (IC_50_ > 10 µM, Supplementary Materials Figure S2).

### 3.7. HSA-EDTA-VO^++^ Penetrates into CNS-1 Glioma Cell Line via Caveolae-Mediated Endocytosis

**Figure 5:**
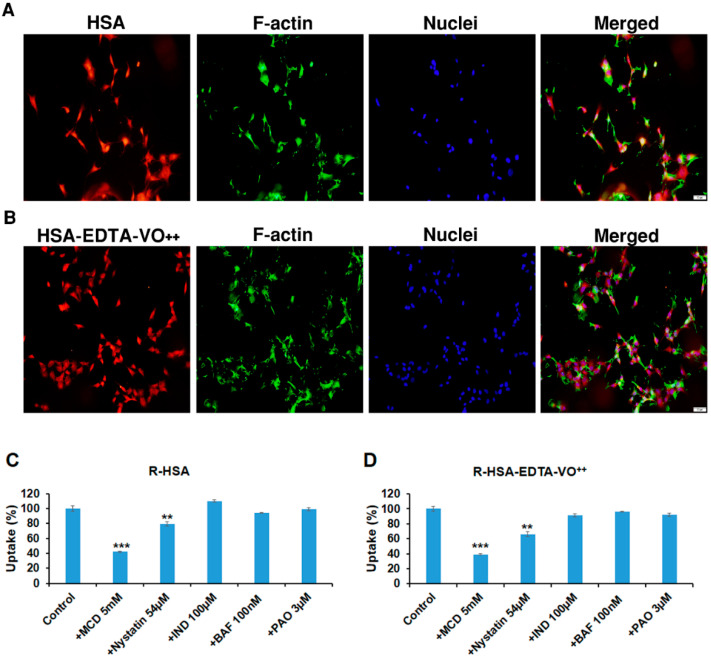
Uptake of HSA and HSA-EDTA-VO^++^ into CNS-1 glioma cell line is a caveolae-mediated endocytosis process. Rhodamine-labeled HSA (**A**) and HSA-EDTA-VO^++^ (**B**) (5 µM) were incubated with the cells for 1 h. The cells were washed, fixed, and mounted on cover slips. The uptake of the compounds was visualized with a fluorescence microscope. The cells were counter stained with phalloidin (green) and Hoechst (blue) for actin filaments and nuclei, respectively. Different blockers were used to determine the mechanism of entry of Rhodamine-labeled HSA (R-HSA) (**C**) and R-HSA-EDTA-VO^++^ (**D**). Data presented as mean ± SEM, *n* = 9 from three different experiments. ** *p* < 0.01, *** *p* < 0.001. Bar, 50 µm.

In order to confirm that this conjugate acts intracellularly, we have prepared rhodamine-labeled HSA and rhodamine-labeled HSA-EDTA-VO^++^ (Experimental part). These compounds were incubated with the cells for varying periods of time. Figure 5A,B show that both HSA and the conjugate were largely taken to the cell interior within 1 h of incubation, indicating that internalization at 37 °C is a rapid event. Uptake of these compounds was already shown after 5 min incubation and also after 24 h (not shown). We then tested a series of inhibitors targeting different endocytosis pathways. Figure 5C,D show that both the native HSA and the HSA-EDTA-VO^++^ conjugate uptake into the CNS1 glioma cells were significantly blocked (58 and 61%, respectively) by MCD, which is a caveolae-mediated endocytosis inhibitor. Nystatin, another caveolae-mediated endocytosis inhibitor also blocked the uptake of HSA and HSA-EDTA-VO^++^ by 20 and 34%, respectively. The clathrin-mediated endocytosis inhibitor PAO had no effect on the uptake of both compounds, nor did the metabolic inhibitor BAF or the caveolae-mediated endocytosis inhibitor IND, which blocks the internalization of caveolae and the return of plasmalemmal vesicles to the cell surface [30].

## 4. Discussion

Vanadium, an anabolic metalooxide in insulin responsive tissues, inhibits a wide variety of phosphohydrolases [31]. As such, it facilitates a variety of biological responses in different directions [32]. In cancer cells, both anti-proliferative and proliferative responses were observed [4]. The question arose whether the anti-proliferative effect of vanadium can be isolated, magnified, and specifically directed toward a tumor cell line. 

In this study, we selected HSA as the protein carrier for obtaining selectivity toward cancer cell lines [15]. Albumin is taken up by malignant tissues as a source of carbon and energy [33,34]. The protein has a single cysteinyl moiety enabling preparation of a monomodified conjugate with MAL-containing compounds [27]. Cysteine-34 is located on the outer surface of HSA distant from the main interior drug binding sites, making it attractive for covalent conjugation of drugs [16]. Derivatization of this moiety with low molecular weight compounds has no or little effect on its native structure and was shown to be efficiently pinocytosed by cancer cells [33,34]. Initially, we treated HSA by one equivalent of DTT, to release disulfide bonded cysteine-34. This procedure yielded HSA having 0.85 ± 0.05 mole-SH/mole protein. Although cysteine-34 is located in a hydrophobic crevice of depth 10–12 Å [35], it reacted with the unbranched MAL-(CH_2_)_2_-NH-CO-EDTA to obtain the macromolecular chelator shown in Figure 1.

Interestingly enough, HSA-EDTA associated with both forms of vanadium at 3–4 fold higher affinity as compared to EDTA or to EDTA-maleimide (Figure 2). Cysteine-34 is positioned in a 10–12 Å deep hydrophobic crevice on the surface of HSA [36]. It therefore appears that the vicinity of this cysteine moiety contributes significantly in elevating the associating affinity of this chelator toward vanadium. PEG30-S-MAL-EDTA showed no higher affinity toward vanadium as compared to that of EDTA (unpublished observation). Both HSA-EDTA-vanadium conjugates studied here were purified on a Sephadex G-50 column prior to analyses for anti-proliferative efficacies. This procedure removed unbound vanadium and vanadium ions that associate with HSA in an EDTA-independent fashion. This purification step demonstrated that the conjugates are stable in the presence of 0.01 M NaHCO_3_ (pH 8.2).

Although not in the frame work of this study, we noted that stable-Sephadex purified complexes of HSA-EDTA with Zn^+2^, Fe^+2^, Mn^+2^, Co^+2^, MOO_4_^−2^, and WOO_4_^−2^ could also be obtained (not shown) despite the fact that Zn^+2^, Fe^+2^, Mn^+2^, and Co^+2^ have considerably lower binding affinities than vanadium toward EDTA [37]. Finally, we demonstrated the superiority of both conjugates in facilitating the anti-proliferative effect in the CNS-1 cell line as opposed to free vanadium (Figure 4). Since the complex of vanadium (+4) with EDTA displays negligible effects on cells, our preference is given to HSA-EDTA-VO^++^.

We refer to HSA-EDTA-VO^++^ as the first example of a possible ‘peripherally non-toxic chemotherapeutically active prodrug conjugate’. Vanadium (+4), a rather anabolic metalooxide, exhibited low peripheral toxicity in rodents and in human diabetic patients [2,38,39,40,41,42]. This is most likely valid for HSA-EDTA, which associates with vanadium (+4) with considerably higher affinity (Figure 2). Reactivation is exclusively an intracellular mediated event and as opposed to other previously studied albumin-drug conjugates [15], the release of the chemotherapeutically active component is a simple intracellular dissociation that takes place half maximally at pH 5.8 ± 0.1 (Table 1). The cytosolic pH of cancer cells is lower than 7.0 [43], suggesting that a sufficient amount of VO^++^ can be released at the cytosol following internalization and more so at the acidic pH of the lysosome [44]. In this context, some tumor cell-related properties may hinder the efficiency of these albumin-vanadium conjugates and should be considered in preclinical and clinical settings. For example, extracellular pH of tumor cells (pHe) is usually mildly acidic [45]. pHe values greatly vary between different tumors and also spatiotemporally within a certain tumor [46]. Thus, certain part of vanadium ions may be released prior to its internalization into the cells depending on the tumor type and location inside the tumor microenvironment. Yet, since the half maximal dissociation value of vanadium (+4) is at pH 5.8 (Table 1), most of the conjugate should remain intact prior to cell uptake.

HSA-EDTA-VO^++^, similarly to native HSA, internalizes into CNS-1 glioma cells mainly through caveolae/lipid rafts-mediated endocytosis (Figure 5). The uptake of both compounds into the cells was blocked in a similar fashion, exhibiting the importance of mono-modification [47]. Two common caveolae-mediated inhibitors, i.e., MCD, and to a lesser extent also nystatin, were the only drugs that significantly blocked the uptake into the cells (Figure 5). MCD and nystatin interfere with the caveolae-mediated endocytosis by binding sterols within the cell membrane. The differences in the magnitude of inhibition between the two drugs might result from their different patterns and/or capacity of sterols binding [48]. It is well documented that native albumin has several pathways to be internalized into cells depending on the cell type and physiological conditions [49]. This includes receptor-mediated endocytosis—a process that is generally blocked by Bafilomycin A1 (BAF)—a specific inhibitor of the vacuolar H^+^-ATPase. H^+^-ATPase localized in the endosomal membrane is responsible for lowering pH inside the endosome, which is an essential process for the dissociation of the ligands and receptors after receptor-mediated endocytosis. Thus, inhibition of vacuolar H^+^-ATPase results in decreased activity of the receptor-mediated endocytosis process. It is reasonable that the CNS-1 glioma cells that originate in the brain, an organ mostly deprived of albumin, do not express receptor/s for albumin, explaining the lack of uptake inhibition by BAF in these cells. Clathrin-mediated endocytosis is also a pathway with which albumin is being internalized into various cells. For example, alveolar epithelial cells internalize albumin via clathrin-mediated endocytosis, but not by the caveolae-mediated pathway [49], demonstrating that albumin internalizes into cells by different pathways depending on the type of the cells and tissue. We also used indomethacin (IND), which blocks caveolae-mediated endocytosis differently than MCD and nystatin by inhibiting the internalization of caveolae and the return of plasmalemmal vesicles to the cell surface [49]. This blocker had no inhibitory effect neither on HSA nor on the conjugate, strengthening the conclusion that caveolae-mediated endocytosis through inhibition of cholesterol-related processes at the cell membrane is the most dominant pathway of HSA and HSA-EDTA-VO^++^ internalization in these cells.

In conclusion, we have engineered a HSA-EDTA shuttling vehicle that can introduce EDTA-associating ligands into a glioma cell line via caveolae-mediated endocytosis, and demonstrated its efficacy to convert vanadium into a powerful anti-proliferative agent. 

## Figures and Tables

**Table 1:** IC_50_ values for the inhibition of acid phosphatase at varying pH values ^1^.

pH	IC_50_ (µM) ^2^	% Dissociated ^3^	Comments
5.0	0.7 ^4^	100	Full dissociation
5.4	1.0	70	
6.0	1.57	44	
7.0	3.7	19	
7.15	5.0	14	
7.31	8.1 ^4^	9	Relatively stable

^1^ IC_50_ values for free vanadium (+4) amounted to 0.7 ± 0.1 µM in all pH tested. ^2^ Assays were carried out for a period of 20 to 60 min with acid phosphatase concentrations of 5 µg/tube at pH 5 to 6 and 25 µg/tube at pH 7–7.31. Reaction was terminated by adding NaOH upon reaching OD_410_ = 0.9 ± 0.1 in the absence of HSA-EDTA-VO^++^. ^3^ Calculated by dividing IC_50_ value of pH 5.0 with the IC_50_ values obtained at each pH measured. ^4^ Valued obtained from Figure 3.

**Table 2:** Anti-proliferative efficacies of HSA-EDTA-vanadium conjugates, and of the building components of those conjugates, in the CNS-1 glioma cell line.

Compound	IC_50_ (µM) ^1^	Potentiation Efficacy (Fold)
Vanadate (NaVO_3_)	5.3	
Vanadyl (VOCl_2_)	8.9	
HSA	>20	
EDTA	>20	
HSA-EDTA	>10	
EDTA·VO_3_^−^ (1:1 complex)	3.2	
EDTA·VO^+2^ (1:1 complex)	16.0	
HSA-EDTA-VO_3_^−^	0.27	19.6
HSA-EDTA-VO^+2^	0.34	26

**^1^** IC_50_ values were calculated from dose response curves using a median-effect plot.

## Data Availability

Data from this study is available from authors upon reasonable request.

## Supplementary Materials

The following are available online at https://www.mdpi.com/article/10.3390/pharmaceutics13101557/s1, Figure S1: Mass spectrometry measurements, Figure S2: Anti-proliferative efficacies of HSA-EDTA-VO_++_ in non-cancer cells, Table S1: Mass spectroscopy; MW calculations of HSA derivatives.

## Abbreviations

BAF: bafilomycin A1; EDTA-dianhydride, diethylentriaminepentaacetic dianhydride; DTT-dithiothreitol; 4.4′ DTDP, 4,4 dithiodipyridine; DIPEA, *N*,*N*-Disopropylethylamine; HSA, Human –serum albumin; HSA-S-MAL-EDTA, a one to one conjugate of HSA in which EDTA is linked to its cysteinyl moiety through MAL(CH_2_)_3_-NH_2_; IND, indomethacin; MAL, maleimide; MAL-(CH_2_)_2_-NH_2_, *N*(2-aminoethyl) maleimide; MCD, methyl β cyclodextrin; Mercapto-HSA, HSA- containing mole cysteine (cysteine-34) per mole HSA; PEG_30_-S-MAL-EDTA, a one to one conjugate of PEG_30_-SH linked to MAL-(CH_2_)_2_-NH-CO-EDTA; PAO, phenylarsine oxide; PNPP, *p*-nihophenyl phosphate.
