# Supplementary material for: Albumin-EDTA-Vanadium Is a Powerful Anti-Proliferative Agent, Following Entrance into Glioma Cells via Caveolae-Mediated Endocytosis
Source: Pharmaceutics. 2021 Sep 25;13(10):1557. doi: 10.3390/pharmaceutics13101557 (PMC8540012; doi:10.3390/pharmaceutics13101557)
Supplement: Supplementary file 1 [file pharmaceutics-13-01557-s001.zip › pharmaceutics-1306656-supplementary.pdf]

pharmaceutics

MDPI

Supplementary Materials: Albumin-EDTA-Vanadium Is a Powerful Anti-Proliferative Agent, Following Entrance into Glioma Cells via Caveolae-Mediated Endocytosis

Itzik Cooper, Orly Ravid, Daniel Rand, Dana Atrakchi, Chen Shemesh, Yael Bresler, Gili Ben-Nissan, Michal Sharon, Mati Fridkin and Yoram Shechter

Table S1. Mass spectroscopy; MW calculations of HSA derivatives [1]

Derivative	MW (Da)	Additional Mass (Da)	Relative Intensities of the Different Sub-Populations (%)
Mercapto-HSA	66,436	–	84.9
HSA-S-MAL-EDTA [lot#7152021]	66,595	+159	55.48
HSA-S-MAL-EDTA [lot#7302021]	66,583	+147	55.47

[1] Masses (in Da) were calculated using the UniDec algorithm. Masses are shown for the most abundant subpopulation in each sample.

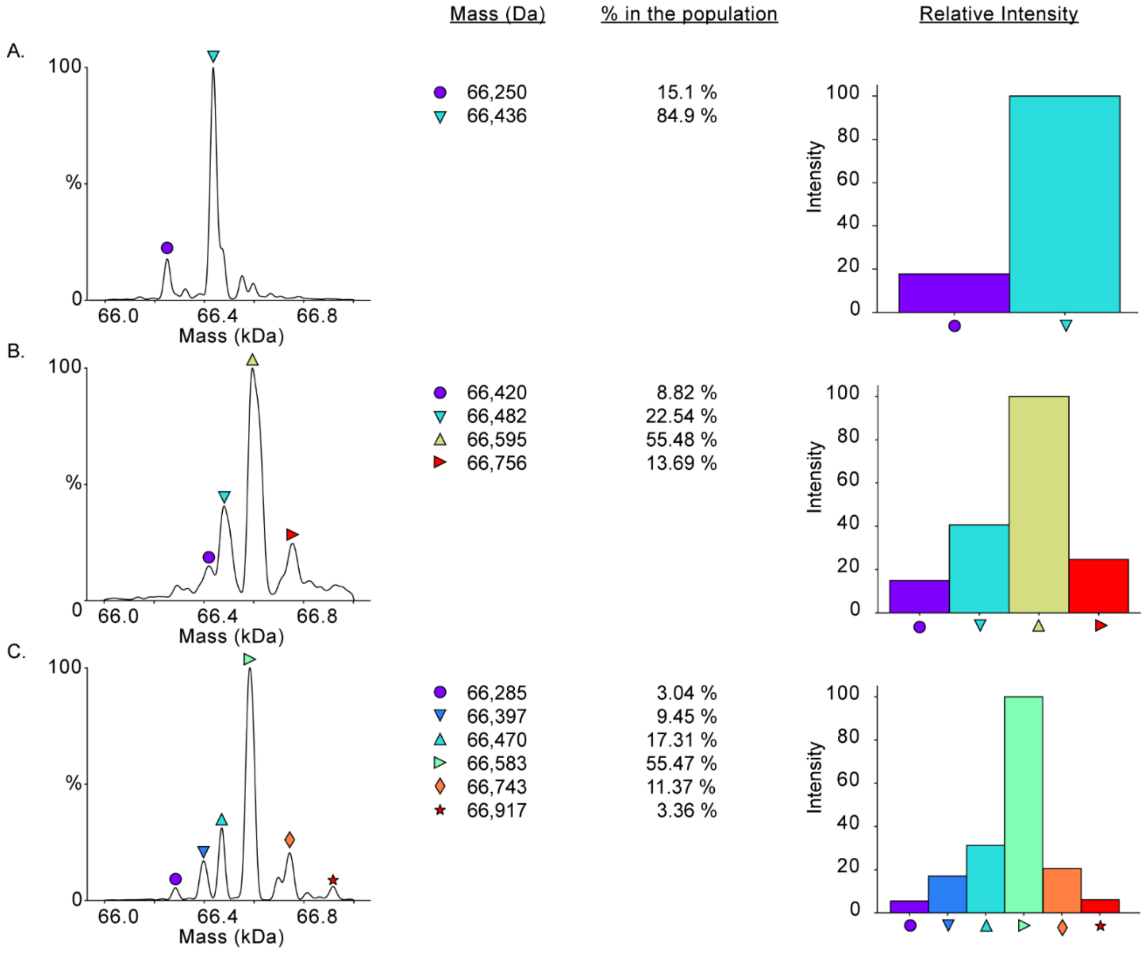

Figure S1. Mass spectrometry measurements. The left panel shows the deconvoluted mass distribution of the samples; (**A**) mercapto-HSA; (**B**) HSA-S-MAL-EDTA [lot#7152021]; and (**C**) HSA-S-MAL-EDTA [lot#7302021]. Measured masses of the different sub-populations, their % in the populations, and their relative intensities are shown on the right.

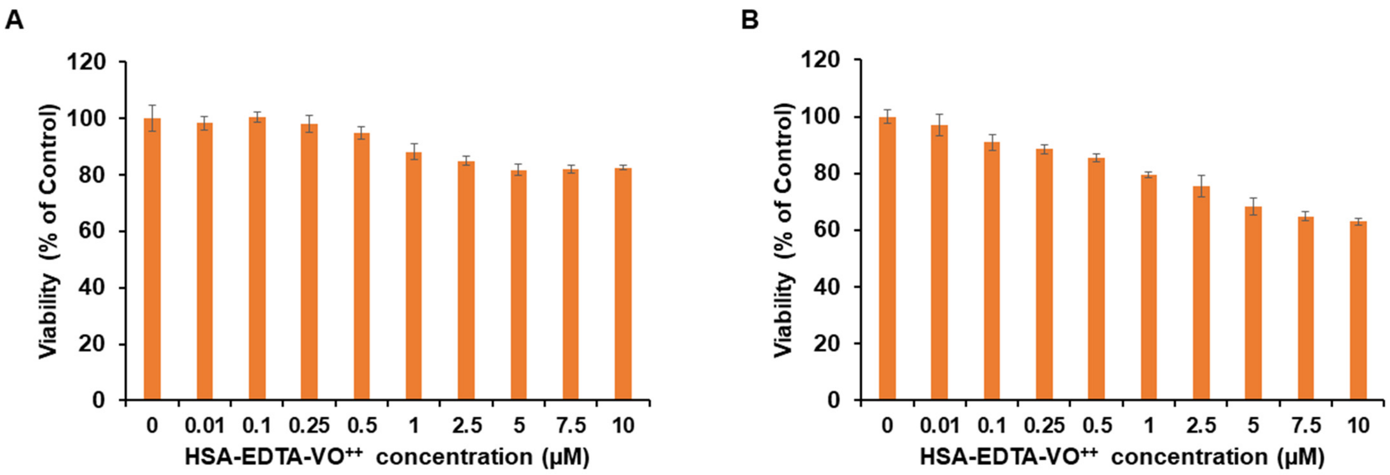

Figure S2. Anti-proliferative efficacies of HSA-EDTA-VO^{++} in non-cancer cells. Dose-dependent toxicity experiments were conducted as described in the Methods section. Human CD34+ endothelial cells (**A**) or primary bovine brain pericytes (**B**) were treated for 72 h with increasing concentrations of HSA-EDTA-VO^{++} in ECM medium before MTT toxicity assay was applied to determine their anti-proliferative efficacies. Experiments were repeated twice with $n = 10$–15. Data are presented as the mean percentage ±SD.
